# HIF2α reduces growth rate but promotes angiogenesis in a mouse model of neuroblastoma

**DOI:** 10.1186/1471-2407-7-139

**Published:** 2007-07-26

**Authors:** Judith Favier, Stéphanie Lapointe, Ricardo Maliba, Martin G Sirois

**Affiliations:** 1 Research Center, Montreal Heart Institute, Montreal, Québec, Canada; 2 Department of Pharmacology, Université de Montréal, Montreal, Québec, Canada; 3 INSERM U772, Collège de France, Paris, France

## Abstract

**Background:**

HIF2α/EPAS1 is a hypoxia-inducible transcription factor involved in catecholamine homeostasis, vascular remodelling, physiological angiogenesis and adipogenesis. It is overexpressed in many cancerous tissues, but its exact role in tumour progression remains to be clarified.

**Methods:**

In order to better establish its function in tumourigenesis and tumour angiogenesis, we have stably transfected mouse neuroblastoma N1E-115 cells with the native form of HIF2α or with its dominant negative mutant, HIF2α (1–485) and studied their phenotype *in vitro *and *in vivo*.

**Results:**

*In vitro *studies reveal that HIF2α induces neuroblastoma cells hypertrophy and decreases their proliferation rate, while its inactivation by the HIF2α (1–485) mutant leads to a reduced cell size, associated with an accelerated proliferation. However, our *in vivo *experiments show that subcutaneous injection of cells overexpressing HIF2α into syngenic mice, leads to the formation of tumour nodules that grow slower than controls, but that are well structured and highly vascularized. In contrast, HIF2α (1–485)-expressing neuroblastomas grow fast, but are poorly vascularized and quickly tend to extended necrosis.

**Conclusion:**

Together, our data reveal an unexpected combination between an antiproliferative and a pro-angiogenic function of HIF2α that actually seems to be favourable to the establishment of neuroblastomas *in vivo*.

## Background

Cellular responses to low oxygen tension are mainly mediated by the activation of heterodimeric transcription factors, called hypoxia inducible factors (HIFs), which consist of a constitutively expressed subunit (ARNT) and an oxygen-regulated subunit, mainly HIF1α and HIF2α (or EPAS1). Under normoxia, HIFα are subjected to enzymatic proline hydroxylation that target them for proteasome degradation via the von Hippel-Lindau (VHL) ubiquitin E3 ligase complex (for review, see [1]).

Both HIFs are overexpressed in many cancer cells and mainly associated with invasiveness, poor prognosis and/or tumour angiogenesis (for review, see [2]). Experimental studies have however led to discrepant results regarding their role in cancer, pointing to functions varying depending on the cell type. Experiments performed with HIF1α-/- embryonic stem (ES) cells conflictingly revealed that HIF1 may act as a positive regulator of tumour growth, most likely through its activation of vascular endothelial growth factor (VEGF) expression [3] or as a negative regulator, possibly through the stabilization of p53 in hypoxic cells [4]. In pancreatic cancer, disruption of HIF1 pathway using a dominant negative strategy reduces tumourigenesis [5]. Opposite results have also been obtained concerning HIF2α. In renal carcinoma cells originating from patients affected by Von Hippel Lindau disease, the abnormal stabilization of HIF2α appears to be directly responsible for tumourigenesis [6,7]. In teratomas derived from ES cells carrying an HIF2α knock-in at the HIF1α locus, Covello et al recently observed an increase in both vascular density and proliferation [8]. In contrast, transfection of HIF2α in a breast cancer cell line inhibits tumour growth, both *in vitro *and *in vivo *[9], while it was recently demonstrated to display pro-apoptotic activities in both rat gliomas and ES cells-derived teratomas [10].

Neuroblastomas are highly vascularized tumours that constitute one of the most frequent solid malignancy of childhood. They arise from immature cells derived from the neural crest and are particularly sensitive to low oxygen tension. Hypoxic treatment of neuroblastoma cells leads to their dedifferentiation towards a neural crest phenotype [11], while overexpression of VHL leads to their differentiation into functional neuron-like cells [12]. Moreover, hypoxia was associated with an increased invasiveness of these tumours *in vivo *[11]. These data point to a role of the HIF pathway in the induction of neuroblastoma's tumour phenotype.

HIF2α plays an important role in the sympathetic nervous system (SNS), both during embryogenesis [13] and in pathological conditions where it is associated with pheochromocytoma malignancy [14]. Hence, it appeared pertinent to assess its role in the SNS-derived model of neuroblastoma C1300. We thus carried out stable transfection of these cells with HIF2α cDNA or with its dominant negative mutant HIF2α(1–485) and show that HIF2α displays antiproliferative but pro-angiogenic activities that favour the establishment of neuroblastoma nodules *in vivo*.

## Methods

### Vector constructions

pCDNA3.1-EPAS1 vector, encoding human HIF2α cDNA (1–870) was a gift from S. McKnight. HIF2α (1–485) cDNA was amplified from pCNDA3.1-EPAS1 plasmid using the elongase polymerase with the following primers: forward, 5'-GCCTCGAGCGACAATGACAGCTGACAAGGAGAAGAAAAGG-3' and reverse, 5'-GCGGATCCTAGCTATTGGGCGTGGAGCAGCTGCTGCT-3', and cloned into PCRII-TOPO (Invitrogen). HIF2α (1–485) cDNA fragment was then recovered by HindIII-XbaI digestion and subcloned into pCDNA3.1 vector (Invitrogen).

### Cell culture and stable transfections

Murine adherent neuroblastoma C1300 cells (clone N1E-115, a gift from N. Lamandé, INSERM U36/U833, Paris, France) were grown in 10 cm petri dishes in DMEM containing 4.5 g/L glucose (Invitrogen) supplemented with 10% foetal bovine serum (Hyclone) and 2% antibiotics; penicillin and streptomycin (Invitrogen). N1E-115 cells were transfected using Lipofectin transfection reagent (Invitrogen), as described by the manufacturer. Transfected cells were selected with G-418 (750 μg/ml; Geneticin; Invitrogen) and cloned by a limiting dilution method.

### RT-PCR

Total RNAs were isolated using the RNeasy extraction kit (Qiagen) and reverse transcribed using random hexamers and the MMLV reverse transcriptase (Invitrogen) as described by the manufacturer. PCR reactions were performed as follows: cDNAs were denatured (94°C for 5 min), submitted to 30 cycles of amplification (94°C for 1 min, Tm°C for 1 min and 72°C for 1 min) and to a final elongation (72°C for 10 min). Primer sequences and Tm used are available upon request.

### Proteins assays

Cells were lysed in Laemmli buffer and 25 to 100 μg of proteins used for Western blot analyses. PVDF membranes were probed with a rabbit polyclonal anti-Tyrosine hydroxylase (1:1000; Institut Jacques Bois), a rabbit polyclonal anti-HIF1α (1:3000, a generous gift from Dr. Darren Richard), anti-phospho-specific Akt IgG (1:1000), anti-phospho-p70 S6 Kinase (Thr389) IgG (1:1000; Cell Signaling Technology) or anti-p42/44 MAPK IgG (1:1000; New England Biolabs). Membranes were subsequently stripped in NaOH and probed with anti-Akt IgG (1:1000, Cell Signaling Technology) and anti-p70 S6 kinase IgG (1:200, sc-230; Santa Cruz). Immunoreactive bands were visualized by enhanced chemiluminescence, digitized using a 2-dimensional gel scanner, and quantified using Quantity One software (Bio-Rad). For HIF2α western blot, proteins were extracted using the NE-PER Nuclear and Cytoplasmic Extraction Reagents (Pierce). 75 μg of nuclear extracts were blotted on PVDF membranes and probed with the ep190b anti-human HIF2α antibody (1:500; abcam).

In another set of experiments, the release of VEGF by native and transfected N1E-115 cells under normoxia and reduced oxygen tension was measured using the murine VEGF development ELISA kit (PeproTech) according to the manufacturer's instruction.

### Cell size evaluation

Cell diameter was evaluated using the Adobe Photoshop software. *In vitro*, cells were seeded at low confluence in 10 cm Petri dishes, in order to avoid the formation of cell aggregates, and grown as described above. For each clone, 10 pictures were taken using a Sony Exwave HAD video camera connected to an Olympus CK2 microscope. In each field, 5 cells were randomly selected and their diameter was measured. This study was repeated once, for a total of 100 cells measured for each clone. For the *in vivo *measurements, the diameter of cells was measured on histological pictures of tissue sections stained with Masson's Trichrome (*see Histology and immunohistochemistry section*). For each clone, 15 cells form 3 different nodules were analysed, for a total of 50 cells per clone.

### *In vitro *proliferation assay

500 000 cells were seeded in 10 cm Petri dishes. After few hours, medium was replaced and the number of surviving cells (T = 0) evaluated by using a Coulter counter (Beckman Coulter). Further counting was performed after 72, 96, 120 and 144 h. The relative proliferation rate of each clone was assessed by calculating the slope of each proliferation curve. This study was performed four times within few weeks of interval.

### *In vivo *tumourigenesis assay

Sixty five 8 weeks-old, female A/J mice, were purchased from Jackson Laboratory and bred under standard conditions at the Experimental Animal Center of our Research center. Tumours were established in accordance to the guidelines set by the Montreal Heart Institute (MHI) animal care committee and the Canadian Council for Animal Protection. Each mouse was submitted to one injection of 2 × 10^6 ^cells in 500 μl Matrigel (Becton Dickinson). Injections were performed subcutaneously at one site on the back of the mouse. The growth of tumour nodules was then measured bidirectionally every 2 days and tumour volume was calculated by the formula π/6 length^2 ^× width. Mice were sacrificed 4 weeks after inoculation. For four HIF2α expressing cells, the MHI ethical committee allowed us to pursue our study up to day 35 post-inoculation.

### Histology and immunohistochemistry

Tumours were resected and fixed for 24 h at room temperature, either in formalin or, for CD31 immunohistochemistry (IHC), in IHC zinc fixative (BD Biosciences Pharmingen). Samples were then dehydrated and embedded in paraffin and six μm-thick sections were submitted to Masson's trichrome staining. Briefly, after treating tissue sections with Bouin's solution to intensify the final coloration, nuclei were stained with Weigert's iron hematoxylin, and cytoplasm and muscle with Beibrich scarlet-acid fuchsin. After treatment with phosphotungstic and phosphomolybdic acid, collagen was demonstrated by staining with aniline blue [15].

Immunohistochemistry was performed as previously described [16] with a rat anti-mouse CD31 (PECAM-1) monoclonal IgG (MEC13.3, BD Biosciences Pharmingen), a rabbit polyclonal anti-proliferating cell nuclear antigen (PCNA) IgG (FL-261, Santa Cruz Biotechnology), or an anti-cleaved caspase-3 (Cell signaling technology).

### Determination of tumour vessel density

The number of blood vessels was quantified on Masson's trichrome stained sections. For each sample, the number of blood vessels was counted in 7 fields of 385 μm^2 ^each, in a blinded manner.

### Statistical analyses

Statistical analyses were performed using the InStat^® ^software. Differences in cell size were evaluated by unpaired t-test and variations in cell proliferation by a paired t-test. *In vivo *growth of tumours and vascular density differences were assessed by a Mann-Whitney test. A p value < 0.05 was considered significant.

## Results

### Establishment and characterization of the cellular models

**Figure 1:**
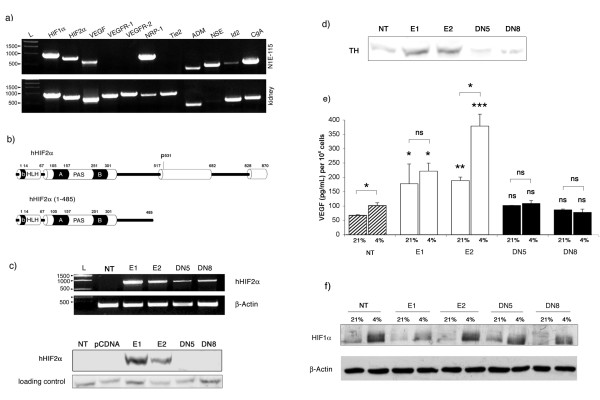
**Characterization of native and transfected N1E-115 cells**. a) Expression of HIF1α, HIF2α, VEGF, Neuropilin-1 (NRP-1), adrenomedullin (ADM), neuronal-specific enolase (NSE), Id2 and Chromogranin A (CgA) as well as HIF2α endothelial-specific targets VEGFR-1, VEGFR-2 and Tie2 in N1E-115 cells and mouse kidney cDNAs. DNA ladder (L) is indicated in base pairs. b) Comparison of the human HIF2α (hHIF2α) 870 amino acids protein with the HIF2α(1–485) mutant shows that both activation domains have been truncated in the dominant negative form. c) Specific RT-PCR amplification of hHIF2α sequence was used to confirm the efficiency of transfection with both hHIF2α (E1 and E2) and hHIF2α(1–485) (DN5 and DN8) expressing vectors. Western blot using a specific anti-human HIF2α antibody confirmed the nuclear presence of the wild-type protein in E1 and E2 clones. d) Tyrosine hydroxylase (TH) expression was assessed by western blot and e) VEGF protein expression quantified by ELISA in duplicate 21% or 4% O_2 _atmosphere. Asterisks on bars illustrate statistical significance compared to NT cells in the same oxygen condition. Upper asterisks indicate significance between 21% and 4% O_2 _for each clone. f) Western blot evaluation of HIF1α levels revealed no variation between the different cell clones in both 21% and 4% O_2_.

We studied HIF2α 's function in the mouse model of neuroblastoma C1300 (N1E-115 clone). To better characterize these cells, we performed RT-PCR experiments to seek for the expression of genes of interest (Fig. 1a). We observed that N1E-115 cells express both HIF1α and HIF2α, their common target genes VEGF and adrenomedullin as well as several neuroblastoma classical markers including neuropilin-1, neuronal-specific enolase, Id2 and chromogranin A.

We then stably transfected N1E-115 cells with plasmids encoding human HIF2α or its dominant negative mutant (DN). HIF2α DN (namely HIF2α(1–485)) comprises the DNA binding domain and the HLH-PAS dimerization domains, but lacks both transactivation domains (Fig. 1b). All commercially available anti-HIF2α antibodies target the C-terminus of the protein, which happens to be deleted in HIF2α(1–485). Screening for transfectants was thus performed by RT-PCR using primers specific to the human HIF2α sequence (Fig. 1c, upper panel). To avoid unspecific responses due to clonal selection, we selected two clones expressing HIF2α (E1 and E2) and two clones expressing HIF2α(1–485) (DN5 and DN8). Western blot experiments using a specific anti-human HIF2α antibody confirmed the nuclear expression of the transfected construct in HIF2α clones (Fig. 1c, lower panel). Untransfected cells (NT) or cells transfected with the empty pCDNA3.1 vector were used as controls (C).

To confirm the activity of HIF2α and HIF2α(1–485) exogenous proteins, we studied the expression of tyrosine hydroxylase (TH), a known HIF2α target gene in the sympathetic nervous system [17]. We observed an increase in TH protein expression in HIF2α clones, and a reduction in HIF2α(1–485) compared to control cells (Fig. 1d). We next incubated the cells in normoxia (21% O_2_) or in low oxygen tension (4% O_2_) for 6 h and evaluated the release of VEGF by ELISA (Fig. 1e). In controls cells, we confirmed that VEGF expression was indeed induced, although moderately, in response to decreased oxygen tension (p = 0,04). As expected, we noted a marked increase in VEGF levels in both E1 and E2 clones, in normoxia (161% (p = 0,017) and 178% (p = 0,012) increase respectively as compared to control cells) and in 4% O_2 _(117% (p = 0,004) and 272% (p < 0,001) increase respectively). In contrast, VEGF protein concentration was not modified in DN5 and DN8 clones in normoxia compared to control cells, while its hypoxic induction was repressed in both clones. Western blot evaluation of HIF1α protein levels confirmed that the differences observed were not due to a deregulation of HIF1α protein expression, but attributable to HIF2α transfections (Fig. 1f).

### Effect of HIF2α on neuroblastoma cell phenotype

**Figure 2:**
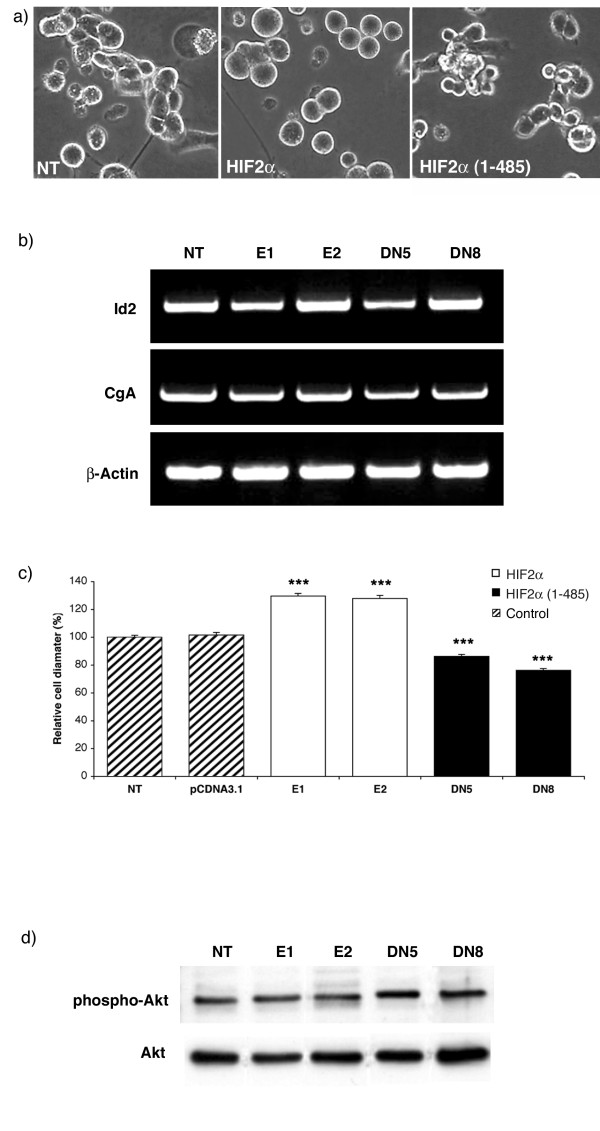
**HIF2α induces neuroblastoma cell hypertrophy**. a) Morphology of control N1E-115 (NT), and N1E-115 transfected with HIF2α and HIF2α(1–485) vectors. b) RT-PCR analyses of neural crest differentiation markers (Id2 and CgA) c) Quantification of the average cell diameter of each clone. Cell size data are mean ± SEM of 100 values for each clone, reported to 100% for NT cells. d) The difference in cell size was not attributable to modification in neither Akt protein expression nor phosphorylation as revealed by Western blot performed on protein extracted from the different clones.

Hypoxia induces an immature phenotype of neuroblastoma cells [11], while inhibition of the HIF pathway by VHL overexpression mediates differentiation toward a neuron-like morphology [12]. We searched for such modifications in HIF2α and HIF2α(1–485) clones, but could not detect any change in the typical cell shape nor in the number of neuronal extension (Fig. 2a). To confirm this observation, we studied the expression of Id2 and CgA markers, which are respectively induced and downregulated during hypoxia-mediated neuroblastoma dedifferentiation [11,18]. Such RT-PCR analyses revealed the absence of any noticeable difference in their expression between the various clones (Fig. 2b).

Unexpectedly, we observed a marked modification in N1E-115 cell size (Fig. 2a and 2c): both HIF2α clones displayed an average 30% increase in cell diameter (p < 0.001), while HIF2α(1–485) cells were 15 to 25% smaller than native or pCDNA3.1-transfected cells (p < 0.001). We performed Western blot experiments to detect the expression and the phosphorylation of two protein kinases, known to play a role in the modulation of cell size [19], i.e. Akt (Fig. 2c) and p70 s6 kinase (data not shown). In both cases, there was no significant difference, neither in protein expression nor in phosphorylation between the various clones.

**Figure 5:**
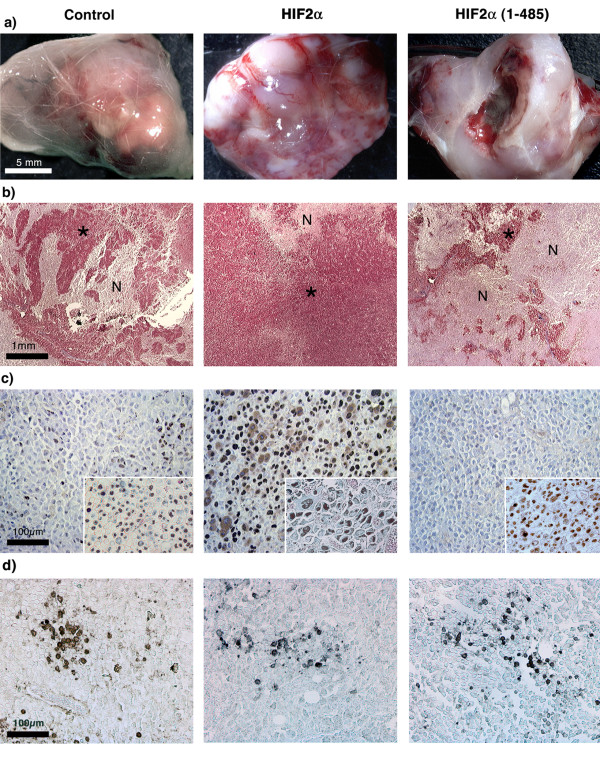
**Effect of HIF2α on tumour necrosis and late proliferation**. a) Macroscopic observations of tumour nodules and b) histological analyses of Masson's trichrome stained sections reveal extended necrotic areas within day 28-HIF2α(1–485) expressing neuroblastomas, compared to control tumours. Such defects are less often observed up to day 35 in HIF2α nodules. c) PCNA immunohistochemistry reveals, within non-necrotic regions (*), a significantly increased number of proliferating cells in HIF2α tumours, while HIF2α(1–485) neuroblastomas present very few labelled cells at day 28. There is no marked difference in proliferation between the three tumour types at day 19 (insets).

It is worth noting that such observations were also performed on histological sections of tumour nodules grown *in vivo (see following experiments and *Fig. 5) where we measured a 22% increase in cell diameter in HIF2α and a 19 % decrease in HIF2α(1–485) nodules compared to control ones (p < 0,001).

### HIF2α reduces neuroblastoma cell growth *in vitro*

**Figure 3:**
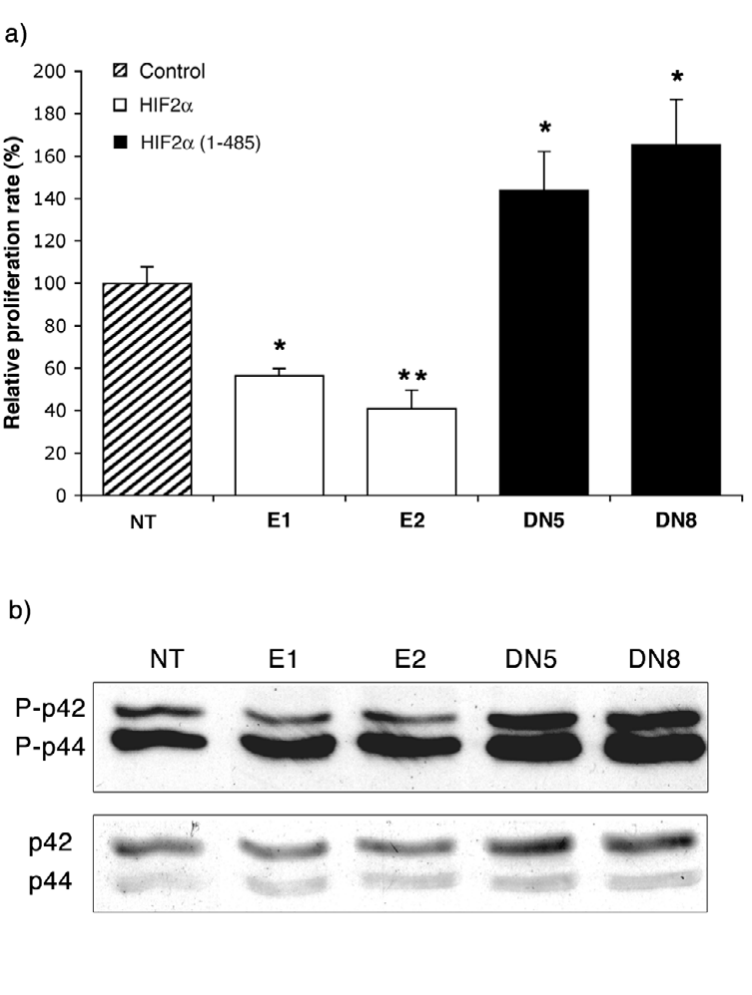
**HIF2α reduces cell proliferation**. a) *In vitro *proliferation assay reveals a reduction in the relative cell proliferation rate of HIF2α expressing cells (E1 and E2) as compared to control cells (NT), while N1E-115 cells transfected with HIF2α(1–485) display an accelerated proliferation (DN5 and DN8). Data are mean ± SEM of 4 independent experiments and reported to 100% for control untransfected cells. *: Significant. b) Western blot analysis reveals an increased in p42/44 MAP kinase phosphorylation in proliferating clones (DN5 and DN8) while the opposite effect is observed in clones displaying a low growth rate (E1 and E2).

The other major change in cell phenotype observed was a modification of proliferation (Fig. 3a). We indeed observed a 44 and 51% decrease in the growth rate of HIF2α cells in E1 (p = 0.015) and E2 (p = 0.004) clones, respectively, while cells expressing HIF2α(1–485) grew 44 and 65% faster than controls in DN5 (p = 0.048) and DN8 (p = 0.039) clones, respectively. During these experiments, we observed no increase in cell death in HIF2α cells compared to control or HIF2α(1–485) clones. The differences in proliferation rates were associated with a decrease in p42/44 mitogen activated protein kinase (MAPK) phosphorylation in HIF2α clones, while this pathway appeared activated in HIF2α(1–485) cells compared to controls.

### Effect of HIF2α on tumour growth *in vivo*

**Figure 4:**
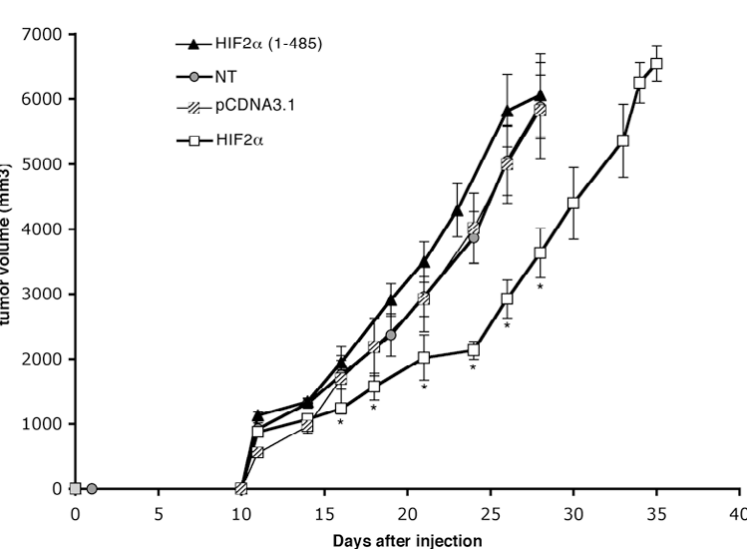
**HIF2α modulates the in vivo growth of neuroblastomas**. The volume of tumour nodules was measured every 2 days after injection of control cells, HIF2α or HIF2α(1–485) transfected cells. Data are mean ± SEM of 15 untransfected tumours (NT), 16 pCDNA3.1-transfected tumours, 18 HIF2α tumours (E1 (n = 9) and E2 (n = 9)), and 16 HIF2α(1–485) tumours (DN5 (n = 9) and DN8 (n = 7)). Each tumour corresponds to one injection in one mouse.

We then evaluated the effect of HIF2α overexpression or inactivation *in vivo *in a tumourigenesis assay in syngenic A/J mice. Cells were injected subcutaneously and the growth of neuroblastoma nodules evaluated over a 4-week period (Fig. 4). As described *in vitro*, we observed that both HIF2α clones and both HIF2α(1–485) clones displayed the same behaviour, respectively. In an attempt to limit the number of animals, we thus pooled the *in vivo *data of E1 and E2 clones, now referred to as HIF2α, and of DN5 and DN8 cells, now referred to as HIF2α(1–485). Control cells formed regularly growing nodules, which were stopped after 28 days, when tumours reached the maximal volume fixed by MHI ethical guidelines. Accordingly with *in vitro *data, we first observed a marked decrease in the tumour growth of HIF2α overexpressing cells when compared to controls. At day 24, the volume of HIF2α nodules was 45% inferior to that of control tumours (p = 0.001). However, from day 24 onward, the growth curve of HIF2α cells displayed a marked acceleration. We thus extended the evaluation period for four HIF2α tumours. We observed that they reached the average size of 28-days control tumours 6 days after them, i.e. at day 34.

Cells overexpressing HIF2α(1–485) did not display the growth expected from the *in vitro *proliferation observations. First, they did grow slightly faster than control cells, to reach a maximal 22% volume increase at day 19 (p = 0.058). From day 19 onward, this divergence however diminished and at day 28, there was no distinction between control and HIF2α(1–485) tumours volumes.

In an attempt to clarify this rather complex *in vivo *effect of HIF2α on neuroblastoma growth, we removed tumours at days 19 (just before the growth acceleration of HIF2α nodules) and 28 for controls and HIF2α(1–485) cells and at days 19, 28 and 35 for HIF2α nodules. Macroscopic examination revealed that 28-days HIF2α(1–485) tumours were pale and highly necrotic compared to controls, whereas equivalent HIF2α tumours (obtained at day 35) appeared particularly dense and red coloured (Fig. 5a). These observations were confirmed by histological observation of Masson's trichrome stained sections. Massive necrosis was observed in HIF2α(1–485) tumours as compared to controls, while HIF2α nodules presented a much-limited number of necrotic regions (Fig. 5b). At day 19, cell proliferation revealed by proliferating cell nuclear antigen (PCNA) immunohistochemistry did not show any difference between the 3 tumour types, which were all actively proliferating (Fig. 5C insets). In contrast, at later stages, we observed that within healthy-looking areas of tumours, the number of proliferating cells was markedly increased in both 28- and 35-days HIF2α nodules compared to controls. In contrast, there was almost no PCNA staining in cells expressing the HIF2α DN (Fig. 5c). This observation does suggest that 28-day HIF2α(1–485) tumours were most probably entering a regression process, while their equivalent 35-day HIF2α counterparts were particularly proliferating. Finally, as HIFs have been described to induce or repress apoptosis, depending on the cell type, we studied the number of cleaved caspase-3 positive cells, outside of the large necrotic areas. In all tumours studied we observed isolated apoptotic cells as well as several areas presenting an increased density of positive cells, but we were unable to use these criteria to discriminate between control, HIF2α and HIF2α(1–485) tumours (Fig. 5d).

### HIF2α promotes tumour angiogenesis *in vivo*

**Figure 6:**
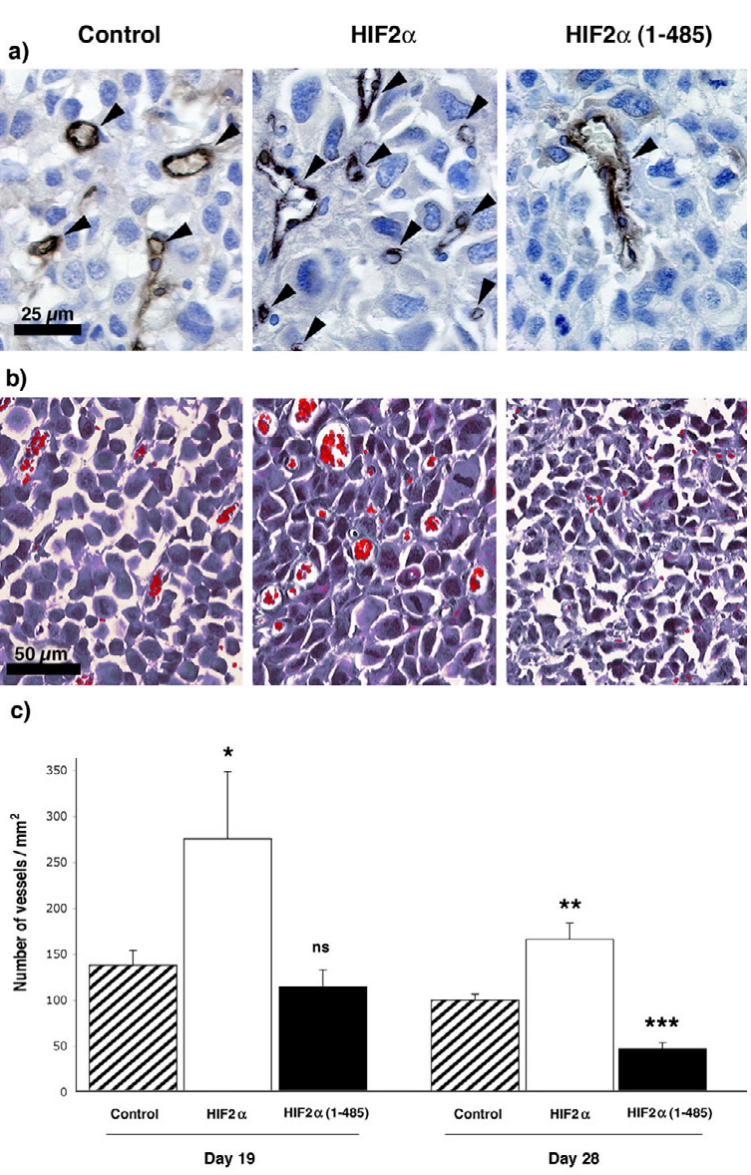
**HIF2α promotes neuroblastoma angiogenesis**. a) Positive staining of endothelial cells in tumours was revealed by PECAM/CD31 immunohistochemistry and b) by Masson's trichrome staining of tumour sections. Both revealed an increase in blood vessel density in HIF2α tumours versus control ones, whereas the expression of the dominant negative HIF2α(1–485) is associated with an apparent inhibition of the angiogenic process. c) Quantification of blood vessel density represents mean ± SEM of 3NT, 3 HIF2α and 4 HIF2α(1–485) at day 19, and of 11 NT, 11 HIF2α and 6 HIF2α(1–485) tumours and day 28.

We hypothesized that the delayed growth of HIF2α tumours and the simultaneous necrosis of fast-growing HIF2α(1–485) nodules could be respectively imputable to an increase and a defect in the angiogenic process. We performed immunohistochemistry analyses to detect the endothelial marker PECAM/CD31, which highlighted a marked reduction in the vascularization of HIF2α(1–485) expressing tumours and an amplification of angiogenesis in HIF2α ones (Fig. 6a). Quantification of blood vessels (Fig. 6c) within healthy regions of each tumour type revealed that vascular density was markedly increased in HIF2α tumours compared to controls nodules (96% increase at day 19, p = 0.05, and 63% increase at day 28, p = 0.006). In contrast, although angiogenesis was comparable between HIF2α(1–485) nodules and control ones at day 19, it then diminished in HIF2α(1–485) to reach a 54% decrease in the number of blood vessels compared to control tumours at day 28 (p = 0.001).

## Discussion

In the present study, we report that HIF2α promotes neuroblastoma cell hypertrophy and reduces their proliferation *in vitro*. However, we also demonstrate that *in vivo*, its antiproliferation function acts in combination with an amplification of the angiogenic process to promote the establishment of well-structured neuroblastoma nodules.

To evaluate the role of HIF2α in neuroblastoma, we overexpressed its native form or inactivated its function using a DN strategy. We used the well-characterized C-terminal deletion mutant HIF2α(1–485), which forms a heterodimer with ARNT, binds to the hypoxia-responsive element (HRE) sequence but lacks the ability to transactivate HRE-driven transcription [20,21]. In light of the parameters studied, we observed reproducible and opposite effects of HIF2α and HIF2α(1–485) respectively, and equivalent results were obtained with two independent clones of each transfectant. These observations strongly argue for the efficiency and the specificity of this approach. However, it is worth noting that HIF2α(1–485) may also prevent HIF1α-mediated transcription [21]. In our model, we thus cannot exclude that HIF2α(1–485) effects may also be partly due to HIF1α inhibition.

We observed a marked effect of HIF2α on the modulation of cell size, which was quantified *in vitro *as well as on histological sections of tumour nodules. The impact of this observation is still unclear, and to the best of our knowledge, such an induction of neuroblastoma cell hypertrophy has never been reported. Interestingly, cellular hypertrophy has been associated with cell cycle arrest in mesangial or vascular smooth muscle cells [22,23]. HIF2α enlarged cells also displayed a reduced proliferating rate associated with a diminution in p42/44 MAPK phosphorylation, while proliferation and phospho-p42/44 were increased in small HIF2α(1–485) cells. Although this is the first report of an antiproliferative role of HIF2α, similar activities of HIF1α have already been described in several cells types [4,24,25]. Consequently, these *in vitro *data led us to conclude that HIF2α would present antitumoral activities in neuroblastoma, as previously proposed in breast cancer cells [9] and gliomas [10]. Our *in vivo *observations led us to reconsider this initial assumption.

We indeed show that overexpression of HIF2α leads to a delayed growth of neuroblastoma nodules, characterized by a well-organized structure and a homogeneous histology 5 weeks after inoculation. In contrast, despite an apparent acceleration in tumour growth, 4 week-old HIF2α(1–485) neuroblastomas present extensive necrosis associated with a marked reduction in the number of proliferating cells within the apparently healthy tumour territories. Due to ethical restriction, we have not studied HIF2α(1–485) tumours beyond this 28-day period. The histological analysis of these tissues, together with the PCNA immunohistochemistry data, were however suggestive of a forthcoming regression, or at least growth arrest, of neuroblastomas expressing HIF2α DN. Hence, the antiproliferative role of HIF2α appears to allow the favourable formation of well-organized tumour nodules instead of reducing neuroblastoma tumourigenesis, as initially assumed. These findings confirm the complexity of HIF2α activity in cancer, which appears to be highly variable depending on the cell-type. In gliomas, Acker et al. observed a comparable negative effect of HIF2α on tumour growth, but an exact opposite phenotype on histology, with increased necrosis in HIF2α tumours and the presence of micronecrotic areas in HIF2α-DN-expressing ones [10].

Finally, we observed that HIF2α transfected cells led to the formation of particularly vascularized tumours. In contrast, we observed that there was a clear defect in the long-term angiogenic progression within neuroblastomas formed by HIF2α (1–485) cells. These tumours were able to initiate a vascularization process, but after four weeks of growth, they presented a marked reduction in vascular density, which could be expected to be responsible for the growth arrest and the important necrosis we observed. The pro-angiogenic role of HIF2α has been established *in vivo *during embryogenesis [26] and in a wound healing assay [27]. In pathological angiogenesis such a role has been suggested by a wealth of expression studies performed on tissue sections, but remained to be clarified in experimental tumour angiogenesis. Recently, discrepant results have been obtained in two models of ES-cells-derived teratomas, demonstrating a positive effect of HIF2α on vascular density [8], or an increase in vessel diameter but no modification of blood vessel density [10]. Our results reveal a pro-angiogenic function of HIF2α by increasing blood vessel density in neuroblastoma, probably mediated by VEGF overexpression. The long-term anti-angiogenic effect of HIF2α(1–485) demonstrates that this transcription factor's endogenous activity is required for an optimal vascularization of such tumours, putatively by improving the integrity and maintenance of the vascular network. These data support a very recently published study, which reported that high HIF2α protein levels were correlated with advanced clinical stage and high VEGF expression and predicted poor prognosis in a clinical neuroblastoma material [28].

## Conclusion

In conclusion, this study confirms the cell-type-dependent heterogeneity of HIF2α 's function in cancer and reveals its unexpected antiproliferative role in the N1E-115 neuroblastoma cell line. Instead of inhibiting cancer progression, this function seems to act in combination with a pro-angiogenic activity of this transcription factor to favour the establishment of appropriately fed, well oxygenized, strong and thus potentially aggressive tumours. These complementary functions, acting together, thus emphasize a favourable role of HIF2α in neuroblastoma tumourigenesis by providing sustained tumour growth, albeit at a slower rate.

## Competing interests

The author(s) declare that they have no competing interests.

## Authors' contributions

JF conceived the study, performed most experiments and wrote the manuscript. SL performed some western blot experiments and participated to cell culture studies. RM carried out the VEGF ELISA assay. MGS participated in the design and coordination of the study and helped to draft the manuscript. All authors read and approved the final manuscript.

## Pre-publication history

The pre-publication history for this paper can be accessed here:


